# Emerging Microbes, Infections, and Spillovers: Charting a Path Forward

**DOI:** 10.3390/v15122392

**Published:** 2023-12-08

**Authors:** Maciej Grzybek, Laura Kakkola, Tarja Sironen, Ravi Kant

**Affiliations:** 1 Department of Tropical Parasitology, Institute of Maritime and Tropical Medicine, Medical University of Gdansk, 81-519 Gdynia, Poland; maciej.grzybek@gumed.edu.pl; 2 Institute of Biomedicine, Faculty of Medicine, University of Turku, 20014 Turku, Finland; laura.kakkola@utu.fi; 3 Clinical Microbiology, Turku University Hospital, 20521 Turku, Finland; 4 Department of Virology, Medicum, University of Helsinki, 00290 Helsinki, Finland; tarja.sironen@helsinki.fi; 5 Department of Basic Veterinary Sciences, Faculty of Veterinary Medicine, University of Helsinki, 00790 Helsinki, Finland

In an age defined by rapid globalization and unprecedented technological advancements, the field of infectious diseases stands at the intersection of complex challenges and promising opportunities [1]. The emergence of new microbes, infections, and spillovers has become an area of critical concern due to the profound impact these phenomena can have on public health and society at large [2]. Recent developments, such as the Ebola and Zika virus outbreaks, as well as the seismic disruption caused by the severe acute respiratory syndrome coronavirus 2 (SARS-CoV-2) pandemic, underscore the urgency of addressing these global threats [3].

Central to these infectious diseases is their zoonotic nature, meaning they can cross species boundaries, often originating in wildlife before reaching human populations [4]. This zoonotic propensity characterizes the majority of emerging pathogens [5]. The dynamics of our interconnected world, marked by globalization and industrialization [6], have significantly altered the vulnerability of both human and animal populations to emerging and re-emerging infectious diseases [7]. This has led to a dramatic evolution in the scale and pace of disease outbreaks, necessitating a proactive approach to understanding and mitigating these threats [8].

As we look forward, it is evident that new infectious diseases will continue to pose a significant and growing risk to global public health in the coming years. 

Global warming significantly influences the rise of zoonotic diseases, as warmer temperatures and altered ecosystems affect the distribution and behavior of wildlife, vectors, and pathogens [9]. The climate change-induced alterations in ecosystems and wildlife habitats increase human exposure to novel zoonotic pathogens. The expansion of vector habitats, changes in vector population dynamics, and the migration of wildlife due to climate change are key factors driving the increase in zoonotic diseases. These changes underscore the need for more integrated approaches to monitor and control these emerging health threats [10].

Zoonotic diseases are primarily driven by microbes, particularly viruses, that are skilled at crossing host species barriers, a phenomenon known as zoonoses, and even establishing human-to-human transmission [11]. Detecting these pathogens and developing effective control measures require a multifaceted approach encompassing the study of reservoirs and vectors, molecular biology, and diagnostic methods.

Given the gravity of the situation, this Special Issue has been convened to delve into the diverse facets of emerging pathogens and viral zoonoses. This collection invited a wide range of contributions, including reviews, research articles, and short communications, with the aim of fostering a deeper understanding of these intricate challenges. The topics to be explored encompass virus discovery, virus–host interactions, pathogenesis, cross-species transmission, virus evolution, reservoirs, and the zoonotic dimensions of these pathogens.

As we navigate the ever-shifting landscape of emerging microbes, infections, and spillovers, this Special Issue serves as a testament to the importance of interdisciplinary collaboration and a call to action for the research community. By addressing the gaps in our knowledge, we can better prepare for the challenges that lie ahead and work towards safeguarding global public health against the inexorable march of emerging infectious diseases.

Now, let us turn our attention to the research articles presented in this Special Issue, which collectively offer crucial insights into the multifaceted challenges we face:

**Swine Influenza Virus Diversity**: The study by Rabalski et al. on swine influenza viruses (swIAVs) in Poland highlights the significance of understanding the genetic diversity of swIAVs, especially considering their potential for reassortment and cross-species transmission. This research, employing next-generation sequencing (NGS), is pivotal for tracking pandemic strains with unprecedented pathogenicity.

**Metagenomics and Zoonotic Surveillance**: Kosoltanapiwat et al.’s research in Thailand showcases the power of metagenomics and advanced molecular techniques to identify unknown viruses, emphasizing the importance of proactive zoonotic surveillance in areas of human–animal interaction.

**Understanding Lumpy Skin Disease**: The study on Lumpy Skin Disease (LSD) by Parvin et al. in Bangladesh provides valuable insights into the clinical manifestations, epidemiology, and pathology of this emerging disease. Genome sequencing helps in enhancing our understanding of the characteristics and epidemiology of LSD in this region.

**Discovery of Kiwira Virus**: Weiss et al.’s discovery of Kiwira virus in African bats demonstrates the importance of monitoring and understanding zoonotic pathogens, particularly in regions where humans and wildlife interact. The potential for spillover to humans raises important concerns.

**COVID-19 Surveillance in Wildlife**: Krupińska et al.’s study evaluating the potential exposure or infection of red deer to SARS-CoV-2 underscores the importance of monitoring potential zoonotic transmission between species.

**African Swine Fever Immune Evasion**: Hong et al.’s exploration of the immune-evasion mechanisms of the African swine fever virus (ASFV) provides crucial insights into how pathogens counteract host immune responses.

**Senecavirus A in House Flies**: Turner et al.’s research on the persistence of Senecavirus A (SVA) in house flies highlights the role of vectors like flies in disease dynamics within specific environments, such as swine farms.

**Mouse Models for COVID-19 Research**: Kant et al.’s development of a mouse model for assessing COVID-19 countermeasures holds significant promise for research on potential treatments and vaccines.

In conclusion, these research articles collectively underscore the need for continuous vigilance and interdisciplinary collaboration in addressing emerging infectious diseases. They emphasize the importance of surveillance, advanced molecular tools, and a deep understanding of host–pathogen interactions. With these insights in mind, we must focus on future research areas.

**Future Research Areas**:

1. **One Health Approach**: The One Health approach, which recognizes the interconnectedness of human, animal, and environmental health, should be at the forefront of future research. Understanding the ecological and behavioral factors contributing to zoonotic spillovers is critical.
2. **Genomic Surveillance**: With the rapid evolution of pathogens, ongoing genomic surveillance is paramount. Identifying key mutations and their implications for host range and transmission will be essential.
3. **Vaccines and Therapeutics**: Developing vaccines and therapeutics for emerging pathogens, including zoonotic viruses, remains a top priority. These should be designed with adaptability to tackle ever-changing viral landscapes.
4. **Vector-Borne Disease Research**: Research into the role of vectors in disease transmission and strategies to mitigate vector-borne diseases will be crucial. This includes both mechanical and biological vectors.
5. **Antimicrobial Resistance (AMR)**: As a complementary challenge to emerging infectious diseases, addressing antimicrobial resistance requires innovative research to develop new antimicrobials and manage existing ones.
6. **Cross-Disciplinary Collaboration**: Fostering collaboration between various scientific disciplines is fundamental for a holistic understanding of emerging infectious diseases. Epidemiologists, veterinarians, ecologists, molecular biologists, and clinicians must work together with social scientists to find solutions to mitigate the impact of emerging infectious diseases.
7. **Public Health Preparedness**: Preparing public health systems to respond rapidly to emerging infectious diseases is imperative. This includes the establishment of robust surveillance networks, risk assessments, and containment strategies.
8. **International Cooperation**: Global cooperation is crucial in tackling infectious diseases that transcend borders. Sharing data, knowledge, and resources is essential to develop collective responses.

As we look to the future, addressing these areas of research will be critical in the battle against emerging microbes, infections, and spillovers. The complexity of these challenges necessitates a holistic, collaborative, and forward-thinking approach [10]. The research articles in this Special Issue underscore our ability to tackle these challenges and the urgency of doing so. By doing this, we can better protect global public health from the evolving threat of emerging infectious diseases.
